# Schizophrenia Detection Using Machine Learning Approach from Social Media Content

**DOI:** 10.3390/s21175924

**Published:** 2021-09-03

**Authors:** Yi Ji Bae, Midan Shim, Won Hee Lee

**Affiliations:** 1 Department of Software Convergence, Kyung Hee University, Yongin 17104, Korea; beasy11@khu.ac.kr (Y.J.B.); midans26@khu.ac.kr (M.S.); 2 Department of Biology, Kyung Hee University, Seoul 02447, Korea

**Keywords:** social media, Reddit, schizophrenia, natural language processing, machine learning, topic modeling, linguistic inquiry and word count

## Abstract

Schizophrenia is a severe mental disorder that ranks among the leading causes of disability worldwide. However, many cases of schizophrenia remain untreated due to failure to diagnose, self-denial, and social stigma. With the advent of social media, individuals suffering from schizophrenia share their mental health problems and seek support and treatment options. Machine learning approaches are increasingly used for detecting schizophrenia from social media posts. This study aims to determine whether machine learning could be effectively used to detect signs of schizophrenia in social media users by analyzing their social media texts. To this end, we collected posts from the social media platform Reddit focusing on schizophrenia, along with non-mental health related posts (fitness, jokes, meditation, parenting, relationships, and teaching) for the control group. We extracted linguistic features and content topics from the posts. Using supervised machine learning, we classified posts belonging to schizophrenia and interpreted important features to identify linguistic markers of schizophrenia. We applied unsupervised clustering to the features to uncover a coherent semantic representation of words in schizophrenia. We identified significant differences in linguistic features and topics including increased use of third person plural pronouns and negative emotion words and symptom-related topics. We distinguished schizophrenic from control posts with an accuracy of 96%. Finally, we found that coherent semantic groups of words were the key to detecting schizophrenia. Our findings suggest that machine learning approaches could help us understand the linguistic characteristics of schizophrenia and identify schizophrenia or otherwise at-risk individuals using social media texts.

## 1. Introduction

Schizophrenia is a severe mental illness that presents with positive (hallucinations, delusions, confused thoughts and disorganized speech) and negative (affective flattening, alogia, and avolition) symptoms [1] and language disturbance [2]. Individuals with schizophrenia are at an elevated suicide risk; a lifetime rate of suicide in individuals with schizophrenia is approximately 10% [3]. Early detection and diagnosis of schizophrenia is challenging, as multiple comorbidities are associated with schizophrenia, complicating the optimal management of patients and potentially limiting positive outcomes [4]. Social media is increasingly used by those with schizophrenia for sharing mental health concerns, connecting with others who have similar mental health experiences, and searching for social support [5]. Textual contents shared on social media platforms offer new opportunities for improving our understanding of self-expressed schizophrenia at an individual and community level. However, much less is known about the topics discussed in online schizophrenia communities and linguistic markers associated with individuals with schizophrenia.

Recent studies have focused on analyzing language from social media posts [6,7,8,9]. With the advent of social media, individuals suffering from mental disorders have found online mental health communities and present their mental health problems with anonymity. Social media platforms provide various forms of mental health communities in which multiple users share their feelings and seek support and advice from other people who may have similar experiences. Online mental health communities within the social media website Reddit, for instance, have become a popular venue for individuals suffering from mental disorders [10]. Reddit supports throwaway and unidentifiable accounts that can protect mental health community members’ identity and allow for self-help support for people struggling with mental health problems. On Reddit, individuals compose anonymous posts on topically focused sub-communities called subreddits and find information about their symptoms and treatment options. Public submissions and their associated comments from mental health subreddits capture the language used by those sharing and processing their mental health experiences online.

Machine learning models have drawn great attention in recent years [11,12]. Machine learning techniques have played an important role in the applications of natural language processing including sentiment analysis, chatbot systems, question answering systems, information retrieve systems, and machine translation [13]. Specifically, the application of machine learning and natural language processing techniques to social media data offers a new lens for the detection of various types of mental illnesses [14,15,16]. Using these techniques, it is possible to identify linguistic markers associated with schizophrenia from social media texts [7,16,17,18,19,20,21,22,23,24]. Prior work has analyzed the Twitter data of self-identified individuals with schizophrenia using machine learning algorithms with linguistic inquiry and word count (LIWC) features [25]. Mitchell and colleagues analyzed a variety of linguistic features of schizophrenia using lexicon-based and open-vocabulary approaches. Further, they classified users affected by schizophrenia from healthy individuals using linguistic features with an accuracy of 82% [18]. Similarly, Coppersmith and colleagues determined language features of various mental health conditions and examined differences in language usage between multiple conditions [19]. Using machine learning and clinical appraisals, Birnbaum and colleagues found that schizophrenia was related to increased use of interpersonal pronouns, especially the third person plurals, and distinguished users with schizophrenia from healthy individuals with a mean accuracy of 88% using LIWC features [7]. Lyons and colleagues used the Reddit data of mental disorders to examine linguistic features related to affective processes and personal pronoun usage and found that schizophrenia was associated with greater use of third person plural pronouns (e.g., they, them) [6]. A recent study using Reddit posts found elevated use of words relevant to health issues, anxiety, negative emotions, and first person singular pronouns among users in schizophrenia compared to healthy controls and also achieved a mean accuracy of 82% in classifying the language of individuals with schizophrenia from that of healthy individuals using LIWC features [17]. These studies demonstrate the potential for machine learning techniques to improve the detection of schizophrenia. However, there remains a significant knowledge gap regarding the linguistic characteristics and the content topics of social media texts related to schizophrenia and their contribution to the detection of schizophrenia.

To address these knowledge gaps, we used a large corpus of social media posts collected from online Reddit sub-communities for schizophrenia (n = 13,156) and control groups (n = 247,569) comprising non-mental health related subreddits (fitness, jokes, meditation, parenting, relationships, and teaching). We extracted LIWC-based linguistic features and content topics within schizophrenia and control groups separately. LIWC was used to analyze texts and obtain linguistic styles, while unsupervised topic modeling was used to characterize the content of text documents. We then compared these results between groups to determine the linguistic differences and the major topics for each group. Using a supervised machine learning approach, we assessed to what extent machine learning classifiers distinguish the language of the schizophrenia group from that of the control group. Further, an unsupervised clustering approach was applied to the features to identify a coherent semantic representation of words in schizophrenia.

The rest of this paper is organized as follows: In Section 2, we describe, in detail, the design of the textual-based schizophrenia detection framework developed for this study. Experimental results are presented in Section 3. Section 4 discusses the results and highlights potential future research directions. Section 5 concludes the paper.

## 2. Materials and Methods

### 2.1. Data Collection

Data was downloaded from Reddit using the Pushshift application program interface [26]. Public posts were collected from the schizophrenia subreddit (r/schizophrenia) to create post data specific to schizophrenia. As a control group, we selected six non-mental health subreddits focusing on positive emotions, exercising, and life (r/jokes, r/fitness, r/meditation, r/parenting, r/relationships, and r/teaching) to ensure that these written posts were not directly related to schizophrenia [9]. For each subreddit, we collected the posts from 23 September 2016 to 23 September 2020. We only included original posts and excluded the comments. We collected titles and bodies of posts along with user IDs and removed posts from bots and ads. This resulted in 60,009 original schizophrenia posts from 16,462 users as well as 425,341 posts of the control group from 248,934 users. We did not seek for approval from the Institutional Review Board because these data were freely available in the public domain and no interactions with the users occurred. Note that personally identifiable information was not included.

### 2.2. Data Preprocessing

For the preprocessing of social media texts, we removed any posts from the control users who wrote posts on the schizophrenia subreddit. We also excluded control posts if the authors disclosed that they currently or previously suffered from any types of mental disorders or if they disclosed therapist or psychologist expenses. We extracted only one post per user to avoid overfitting [15]. After each title was concatenated with its corresponding bodies, all capital letters were converted to lower case and posts were then tokenized to split the sentences into words (tokens). We removed unnecessary punctuation, numbers, and stopwords using the natural language toolkit (NLTK) [27]. Note that we removed specific keywords “schizophrenia” and beginning with “schizo” for each subreddit for the classification tasks. We performed the lemmatization and stemming steps to reduce each word, respectively, to its lexical form and to return only the base of a word using the NLTK package [27]. The final post datasets consisted of the schizophrenia group with 13,156 posts as well as the control group with a total of 247,569 posts (Table 1; additional details in Supplementary Table S1 and Figure S1). These text datasets were used subsequently for linguistic analysis, topic modeling, classification, and unsupervised clustering.

### 2.3. Linguistic Inquiry and Word Count (LIWC) Analysis

The linguistic features were extracted using the LIWC package [28] and the *liwcalike* function from the quanteda package [29]. We assessed the structural and psychological components of the text based on psychometrically validated dictionary, word stems, and emotions assigned to a range of categories [28]. In the present study, we extracted a total of 22 LIWC features for each post, namely linguistic processes (word count and words more than six letters), function words (personal pronouns, first person singular, first person plural, second person, third person singular, third person plural, and impersonal pronouns), time orientations (past focus, present focus, and future focus), and psychological processes (positive emotion, negative emotion, anger, fear, joy, disgust, sadness, anticipation, trust, and surprise) [28].

We compared the linguistic features between the schizophrenia and the control (non-schizophrenia) groups. The D’Agostino and Pearson’s test (α = 0.05) were conducted to test whether each of the linguistic features was normally distributed. As data followed a normal distribution, a two-tailed t-test was performed to determine whether the linguistic features differed between groups. The threshold of statistical significance was adjusted using the false discovery rate (FDR) method to correct for multiple comparisons. All *p*-values remained significant at *p* < 0.05.

### 2.4. Topic Modeling and Analysis

Topic modeling was then conducted using the Latent Dirichlet Allocation (LDA) [30] implemented in the *gensim* library, which captured sets of words that typically appeared in posts across schizophrenia and control subreddits. The LDA model returned the probability distributions of words to determine the topics that are represented in a collection of textural data. Each of the topic distributions was then used as a feature value equal to the probability of that topic for topic analysis and classification. We tested multiple LDA models to assess topic stability. A final LDA model with 10 topics was selected to ensure distinct and important topics for each subreddit, separately. We then manually assessed common themes across posts for each subreddit.

To compare topic distributions between schizophrenia and control groups, an LDA model with 10 topics was created based on the schizophrenia posts. This schizophrenia LDA model was then applied to all control subreddits to assess the distribution of the control posts across the schizophrenia topics. Comparison of topic distributions between schizophrenia and control groups was performed using a two-tailed *t*-test (α = 0.05) with the FDR correction to test whether the incidence of these topics differed between the two groups. All p-values remained significant at *p* < 0.05.

### 2.5. Classification

We assessed to what extent machine learning classifiers distinguish the posts from the schizophrenia subreddit from those of the control subreddits using topic distributions and LIWC features. Binary classification was performed on the schizophrenia group (r/schizophrenia) versus a control group consisting of six non-schizophrenia subreddits (r/jokes, r/fitness, r/meditation, r/parenting, r/relationships, and r/teaching). Posts from the control group were randomly downsampled to create a balanced dataset (n = 13,156 posts for each group). The final control group consisted of 5186 posts from r/relationships (39%), 4429 posts from r/jokes (34%), 1530 posts from r/fitness (12%), 1246 posts from r/parenting (9%), 603 posts from r/medication (5%), and 162 posts from r/teaching (1%). We only used one post per user to avoid overfitting for each group. This balanced dataset (26,312 × 42 matrix) was randomly divided into a training set (80%) for model training and a testing set (20%) for model evaluation. We evaluated four different algorithms, namely support vector machine, logistic regression, naive Bayes, and random forest [31], which are commonly used because they are interpretable and resilient to overfitting [32]. The four different algorithms were applied to the same feature dataset. For each algorithm, we tuned hyperparameters using 10-fold cross-validation to learn the model parameters and evaluate the model. Each algorithm was trained using grid search to find the parameters that give the highest accuracy. With this technique, we built a model for each possible combination of all of the hyperparameter values provided, evaluating the model, and selecting the model that produced the best results. The performance of each algorithm was quantified by recall = true positives/(true positives + false negatives), precision = true positives/(true positives + false positives), F1-score = 2 × (precision × recall)/(precision + recall), and accuracy = (true positives + true negatives)/(true positives + true negatives + false positives + false negatives) [7,15,33,34]. We then determined important features to understand how distinct languages manifest in the schizophrenia group using the Shapley additive explanation (SHAP) approach [35], which estimated a Shapley value for each feature to measure the importance of a set of features.

### 2.6. Unsupervised Clustering

We performed unsupervised clustering to test whether there were coherent semantic groups of words in schizophrenia on the basis of the features used for classification. To do this, we chose to use the term frequency–inverse document frequency (TF-IDF) to represent posts as feature vectors, as they are amongst the most widely used features in computational linguistic studies [7,9,15]. TF-IDF measures how important a particular word is with respect to a document and the entire corpus. Using the TF-IDF vectorizer in the scikit-learn library, each post in the schizophrenia subreddit was represented as a feature vector of normalized TF-IDF scores of the top 1024 unigrams (i.e., a sequence of one word in a sentence). Posts from the control subreddits were processed through the same processing pipeline. Among the classifiers tested, we chose logistic regression due to its relative simplicity and ability to capture features important for classification. Using these features (26,312 × 1024 matrix), we first trained an L1-penalized logistic regression with the regularization strength parameter (C = 0.1) to perform a binary classification (schizophrenia versus control groups) from which the 100 most predictive features associated with the top positive regression weights were selected. Second, we represented each word using the document vector, which is thought to be a semantic representation of the word [15]. Third, the feature set was reduced to five principal component analysis (PCA) components, which were successfully used to reduce the number of features in the data [9,15]. PCA was chosen because it removes correlated features, improves the performance of machine learning algorithms, and helps in overcoming the overfitting issues [31,36]. Fourth, we reduced the dimensionality of the semantic space to two dimensions using t-distributed stochastic neighbor embedding (t-SNE) with a perplexity of 7 for choosing the optimal number of clusters as well as ease of visualization [37]. t-SNE can capture nonlinear structures in the data and preserve the local and global structure of the data [37]. Dimensionality reduction using t-SNE paved the way for subsequent clustering analysis. Finally, we applied an unsupervised learning technique of DBSCAN to the most predictive words using an epsilon of 0.3 and minimum samples of three [15,38] in order to identify coherent semantic groups of words in schizophrenia. The DBSCAN clustering algorithm was chosen because it finds clusters by identifying dense groups of points in the data, making few assumptions about the distributions of these groups and no assumptions about the number of clusters [38]. Out of 20 t-SNE iterations, we retained eight semantic clusters as it provided the most semantic characterization of words in schizophrenia. We confirmed the validity of cluster annotation through post review.

To confirm the reliability of the unsupervised clustering results, we created the training datasets by randomly resampling the half of the posts 100 times and performed the classification tasks on the training sets. We then repeated the same unsupervised clustering analysis on the regression weights estimated from the half of the sample. See Supplementary Materials for more details.

## 3. Results

### 3.1. Linguistic Inquiry and Word Count (LIWC)

**Figure 1:**
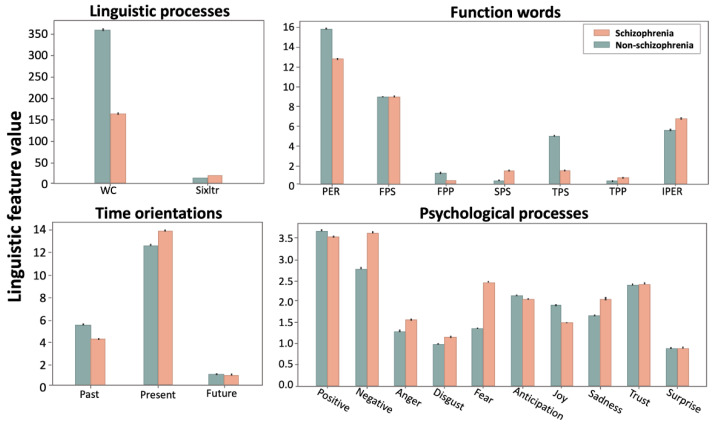
Comparison of the linguistic features between the schizophrenia and the non-schizophrenia (control) groups. Bars and error bars correspond, respectively, to averages and standard deviations across the linguistic values for each linguistic feature. WC = word count; Sixltr = words longer than six letters; PER = personal pronouns; FPS = first person singular; FPP = first person plural; SPS = second person singular; TPS = third person singular; TPP = third person plural; IPER = impersonal pronouns.

Pairwise comparisons show that schizophrenia had lower use of word count (WC) (t = −74.1; *p* < 0.001) and higher use of words longer than six letters (t = 53.7; *p* < 0.001) than the controls (Table 2). The use of first person plural pronouns (t = −76.5; *p* < 0.001) and third person singular pronouns (t = −101.9; *p* < 0.001) was statistically lower in schizophrenia than the controls, whereas the occurrence of second person pronouns (t = 37.9; *p* < 0.001), third person plural pronouns (t = 24.3; *p* < 0.001), and impersonal pronouns (t = 31.1; *p* < 0.001) was statistically higher in schizophrenia than the controls. In time orientation words, we found a decreased use of past focus (t = −34.7; *p* < 0.001) and an increased use of present focus in schizophrenia (t = 24.4; *p* < 0.001), with future focus being comparable between groups (*p* > 0.05). We also found a greater use of affective process words corresponding to negative emotion, anger, fear, disgust, and sadness (all *p* < 0.001), as well as a decreased use of words corresponding to positive emotion, joy, and anticipation (all *p* < 0.001) in schizophrenia compared to the controls. The use of trust and surprise words was comparable between the schizophrenia and the control groups (all *p* > 0.05). The details of the pairwise comparison results are shown in Table 2 and displayed in Figure 1.

### 3.2. Topic Detection and Comparison

**Figure 2:**
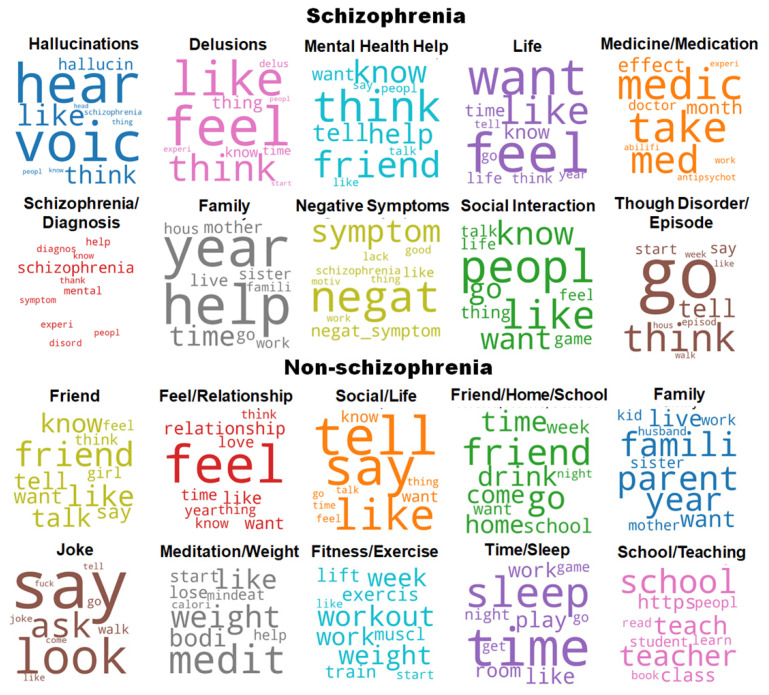
Word cloud visualization based on the distribution of words for each topic discovered from the schizophrenia and non-schizophrenia (control) groups.

Topics extracted from the schizophrenia and the non-schizophrenia (control) subreddits are listed in Table 3. Word clouds in Figure 2 show the most common words in each topic for the schizophrenia and non-schizophrenia subreddits. Topics extracted from the LDA model on the schizophrenia subreddit largely corresponded to the expected topics, including the schizophrenic symptoms and medication (Table 3). Topics emerged related to “schizophrenia and diagnosis” and “medicine and medication”. Topics related to the symptoms of schizophrenia largely appeared, which included “hallucinations”, “delusions”, “negative symptoms”, and “thought disorder and episode”. More general topics such as “social interaction”, “life”, “family”, and “mental health help” also emerged, which captured common topics discussed in the schizophrenia subreddit. Compared to the schizophrenia subreddit, the control subreddit posts had far fewer topics about medication and specific symptoms (Table 3). As expected, the dominant topics discussed in the control subreddits were “family”, “meditation”, “relationships” “life”, “school”, and “fitness”, corresponding to non-mental health related subreddits.

**Figure 3:**
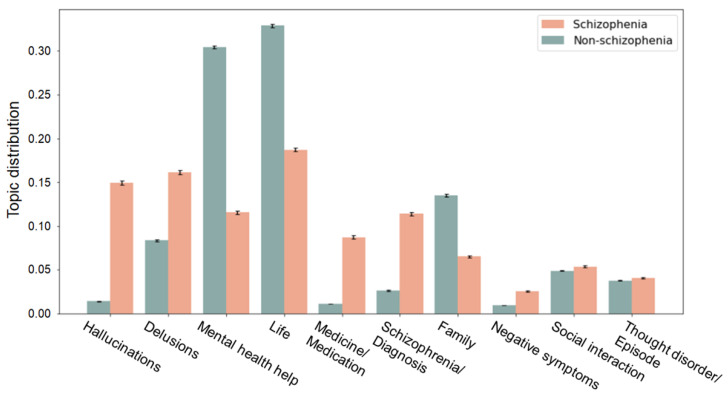
Comparison of the topic distributions between the schizophrenia and the non-schizophrenia (control) groups within topics identified from the schizophrenia posts. Bars and error bars correspond, respectively, to averages and standard deviations across the topic distribution values. All pairwise comparisons show significant differences in topic distributions between the two groups. Schizophrenia has statistically higher topic distributions in “hallucinations”, “delusions”, “negative symptoms”, and “medicine and medication” (all *p* < 0.001) and lower values in topics of “life” and “family” (all *p* < 0.001), compared to the control group.

Figure 3 shows the distribution of topics in schizophrenia and non-schizophrenia subreddits over the 10 topics extracted using LDA. Across topics identified from the schizophrenia group, FDR-corrected pairwise comparisons show that schizophrenia had higher topic distributions in several specific topics such as “hallucinations”, “delusions”, “negative symptoms”, and “medicine and medication” (all *p* < 0.001) and lower values in topics of “life” and “family” (all *p* < 0.001), compared to the control group (Figure 3).

### 3.3. Classification

**Figure 4:**
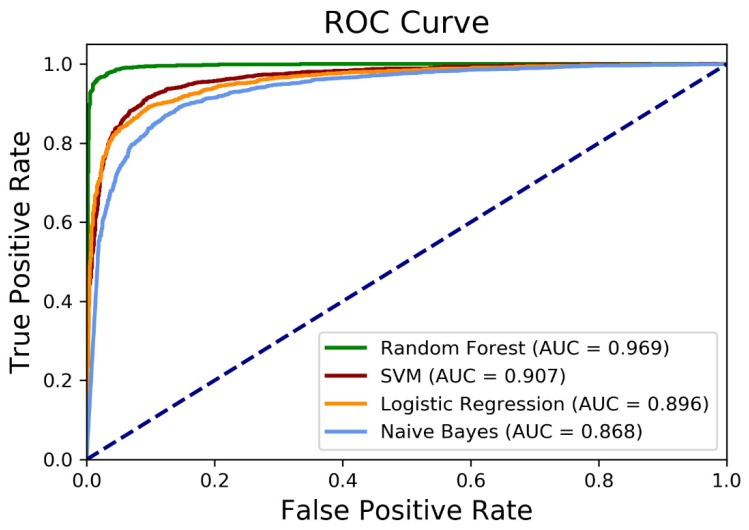
Receiver operating characteristic (ROC) curves for the classification. AUC = area under the ROC curve. Additional details are provided in Supplementary Table S2.

The performance of each algorithm is listed in Table 4. Among algorithms tested, the random forest algorithm achieved the highest accuracy of 96% (recall = 94%, precision = 98%, F1-score = 96%), which exceeds all other accuracy results (86–91%) with the other three algorithms. Similarly, the random forest model performed best with the area under the receiver operating characteristic (ROC) curve (AUC) of 0.97, whereas the other three models provided lower performance with an AUC range of 0.87–0.91 (Table 4; Figure 4). Additional details on the algorithm performance based on the input features are provided in Supplementary Table S2.

**Figure 5:**
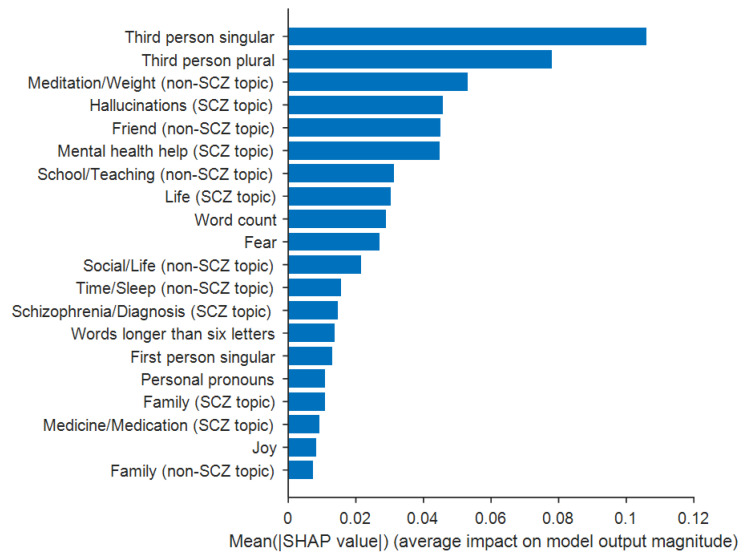
A plot of Shapley values for each feature. Shown is the SHAP feature importance quantified as the mean absolute Shapley value. SCZ = schizophrenia; non-SCZ = non-schizophrenia. Additional details are provided in Supplementary Table S3.

The random forest model was chosen for further analysis (i.e., feature importance) given that it had the greatest classification accuracy. For each feature, the SHAP feature importance quantified as the mean absolute Shapley value is shown in Figure 5. The feature of “third person singular pronouns” was the most important feature and the feature of “family” from the non-schizophrenia topic was the least important feature. Additional details on the feature importance are provided in Supplementary Table S3.

### 3.4. Unsupervised Clustering

**Figure 6:**
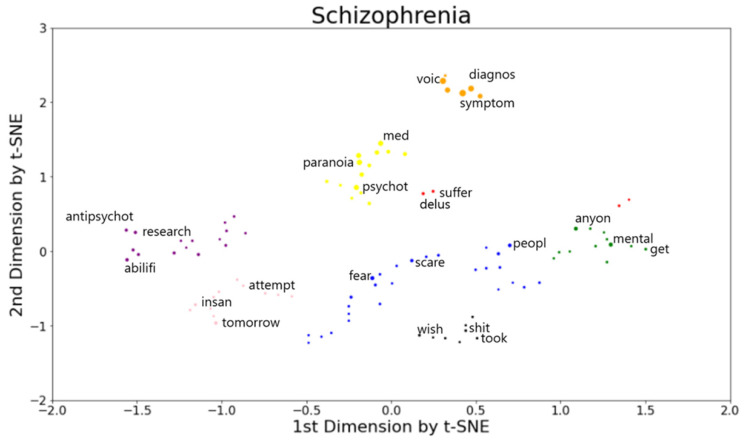
Text plot of words that distinguish the language of the schizophrenia group from the non-schizophrenia (control) group. The 100 most predictive words for schizophrenia are shown. Words are projected in a 2-D space based on their document vectors, after dimensionality reduction with t-SNE. Colors indicate clusters assigned by DBSCAN. In each cluster, the top three most predictive words are labeled. The marker size is also scaled linearly with predictive rank, where larger markers indicate more predictive words.

Figure 6 shows coherent semantic clusters formed based on the top 100 predictive words, out of which the top 10 weighted features obtained from the logistic regression classifier are shown in Table 5. Word positions were determined after dimensionality reduction of the word embedding (TF-IDF features with unigram) and clustering the positions using DBSCAN (Figure 6). Table 6 lists the words associated with each of the semantic clusters shown in Figure 6. The clusters were ordered in terms of their average predictiveness of schizophrenia, from most to least predictive. The words of schizophrenia were also ordered by predictiveness within each cluster. The most predictive cluster (orange cluster) included groups of words mostly related to symptoms of schizophrenia such as “voice”, “hallucinations”, and “hear”. The second and third predictive clusters (yellow and red clusters) had specific symptom-related words such as “psychotic”, “paranoia”, and “delusions”. There was a cluster (blue cluster) forming negative sentiment words such as “fear”, “scare”, and “afraid”. Other clusters included the words related to support (purple cluster) such as “antipsychotic”, “research”, “profession” and “medicine” as well as mental health help (green cluster) such as “mental”, “ill”, and “thank”, and “wonder”. These findings remained consistent when (1) TF-IDF features along with bigram vectorization was used or (2) the half of the posts was used for unsupervised clustering. See Supplementary Materials for more details.

## 4. Discussion

The present study leveraged social media data collected from social media platform Reddit over the course of about four years to test whether the signs of schizophrenia are present in people’s natural language. We applied natural language processing and machine learning techniques including statistical analysis of LIWC linguistic features, supervised learning, and unsupervised learning in order to identify linguistic makers of schizophrenia from social media content.

### 4.1. Linguistic Characteristics of Schizophrenia

We undertook a comprehensive linguistic characterization of schizophrenia and non-schizophrenia (control) posts. By comparing to the frequencies of each LIWC linguistic feature extracted from the control subreddits, we found marked linguistic differences between the posts written by those expressing schizophrenia-related mental issues and control participants (i.e., people who post on non-schizophrenia subreddits). These differences spanned various linguistic categories including linguistic processes, function words, time orientation, and psychological processes. We found that schizophrenia was associated with lower use of word count, first person plural (e.g., we, our), third person singular (e.g., s/he, hers, him), past tense, and positive emotion words. Conversely, people who participated in the schizophrenia subreddit had a higher use of second person (e.g., you), third person plural (e.g., they, them), impersonal pronouns (e.g., one, it), present tense, and negative emotion words. It has been suggested that decreased use of word count is associated with negative symptom severity assessed using the Positive and Negative Syndrome Scale (PANSS) [39]. Pronouns indicate whether an individual’s focus is on the self (first person singular), on others (second person), or on the self as part of a social group (first person plural) [6]. The present results are aligned with several linguistic indicators of schizophrenia in previous studies that found that the users of the schizophrenia group used first person plural and third person singular pronouns less often than the control group [17]. The lower use of first person plural pronouns in schizophrenia involves decreased self-focus and social disaffiliation and withdrawal [6,40]. Consistent with previous research [6,7,17,18,19], users with schizophrenia used more third person plural pronouns than the controls, which may reflect an externalizing bias, paranoid thinking, and persecutory delusions associated with schizophrenia [6,40]. We also replicated a greater use of negative emotion words (e.g., anger, fear, sadness) in schizophrenia [6,18,41] associated with poorer theory of mind and greater hostile or aggressive attributional style [39].

Many of our findings were consistent with previous studies, whereas some of the linguistic features identified differed from prior work. We found only few prior studies that used social media texts exclusively from the schizophrenia subreddit on Reddit for assessing LIWC linguistic features associated with schizophrenia [6,17]. It is perhaps not surprising that there exist discrepancies with regard to linguistic differences, since those studies used social media data from other online discussion forums (e.g., Twitter) [7,18,19] and analyzed fewer linguistic features [6]. Furthermore, the choice of subreddits for creating a control group differed [6,17], thereby introducing variations in linguistic features and confounding inter-study comparisons when identifying linguistic markers of schizophrenia. Nevertheless, our results suggest that linguistic characteristics of schizophrenia identified may help to facilitate studies of this kind to identify the social media markers of schizophrenia

### 4.2. Topic Detection and Comparison

Using unsupervised LDA approach, we extracted the topics discussed among individuals in schizophrenia and non-schizophrenia (control) subreddits, separately. As expected, the schizophrenia subreddit was found to discuss the nature of this disorder and medication. The topics representing the symptoms of schizophrenia emerged from the schizophrenia subreddit, namely “hallucinations”, “delusions”, and “negative symptoms”. Individuals with schizophrenia often have positive symptoms (hallucinations and delusions) associated with altered perceptions, abnormal thinking, and odd behaviors as well as negative symptoms related to loss of motivation, social withdrawal, and difficulty expressing emotions [42]. Content topics corresponding to “diagnosis”, “medicine”, and “medication” were also more common in the schizophrenia subreddit. In comparison to the schizophrenia posts, it is not surprising that prominent topics such as “family”, “social”, “friend”, and “relationships” were more common in the control subreddits. There was also correspondence between the topics discovered such as “family” and “life”. This indicates that the topics discussed in schizophrenia extend beyond schizophrenia-related symptoms and medication [9,18]. Further, pairwise comparison results showed that significant differences between the two groups were found in specific symptom-related topics (“hallucinations”, “delusions”, and “negative symptoms”) (Figure 3). Our results indicate that distinct topics differentiated between the schizophrenia group and the control group by means of natural language processing and unsupervised LDA techniques. The present results suggest that both increased use of symptom-related words and decreased occurrence of positive general topics are indicative of language in schizophrenia.

### 4.3. Classification and Feature Importance

We formulated four different binary classifiers to examine the usefulness of the extracted features (topic distributions and LIWC features) in distinguishing posts of the schizophrenia group from those of the control group. We showed that topic distributions and LIWC features could be used to detect schizophrenia with a high degree of accuracy (86–96%). We found that the machine learning model based on a random forest algorithm achieved the highest accuracy of 96% (recall = 94%, precision = 98%, F1-score = 96%, and AUC = 0.97) in distinguishing between the schizophrenia and the control groups, while the other algorithms performed similarly with classification accuracies between 86% and 91% (recall = 87–91%, precision = 82–90%, F1-score = 89–93%, and AUC = 0.87–0.91). These findings outperformed classification accuracies for the schizophrenia posts reported in prior studies. Using linguistic data of Twitter users with schizophrenia, a random forest classifier performed with a mean accuracy of 88% [7]. A logistic regression classifier distinguished Reddit users with schizophrenia from control users with a mean accuracy of 82% on the basis of LIWC features [17]. A support vector machine with LIWC features and topic distributions classified schizophrenia sufferers with an accuracy of 82%. The classification accuracies in our study are also comparable with those reported for other mental disorders such as depression (70%) [43], anxiety (98%) [44], and bipolar disorder (87%) [45].

By applying the SHAP approach to the random forest model that outperformed the other models, we established features key to distinguishing schizophrenia posts from control posts, which helps understand to what extent different features contribute to the classification of the schizophrenia posts. The most important features used to classify between the two groups were “third person singular pronouns” and “third person plural pronouns”, consistent with the LIWC feature analyses such that these features had the highest differences between groups (Table 2). The features least contributing to the classification were “joy” and “family” variables. These results suggest that LIWC features and topic distributions extracted from social media data aid in the detection of schizophrenia with a high degree of accuracy.

### 4.4. Semantic Characterization of Schizophrenia

We established coherent semantic clusters that represent important conversation words in the schizophrenia subreddit through supervised and unsupervised learning techniques. Consistent with major symptoms of schizophrenia [42], an unsupervised clustering analysis revealed prominent symptom-related clusters such as “hallucinations”, “paranoia”, and “delusions”, indicating that people who participated in the schizophrenia subreddit tend to discuss their schizophrenia symptoms. The clusters included negative sentiment words such as “fear”, “scare”, and “afraid”, indicating that individuals tend to express their feelings and health anxiety about schizophrenia via social media. People also seek support and advice from other people as indicated in the clusters by “antipsychotic”, “research”, and “profession”. Our results suggest that unsupervised clustering methods applied to the features, estimated from supervised classifiers, reveal important language markers of schizophrenia in social media users. Furthermore, the machine learning models used in our study provide a means for clinicians to monitor symptoms more effectively and the potential for early detection of schizophrenia. This would be beneficial to young people and first-degree relatives of schizophrenia patients who are prodromal (clinically appearing to be at high risk for schizophrenia). Early identification of individuals at clinical risk for psychotic illness is critical for early intervention. Efforts at early identification, therefore, could benefit from machine learning models proposed in our study.

### 4.5. Methodological Considerations

We acknowledge several limitations that could be addressed in future studies. The focus of this study was on the methodological aspects of identifying specific textual information in schizophrenia, not on the examination of various types of mental disorders. The present methods can be straightforwardly applied to other text data involved in different forms of mental illnesses in future studies [8,15]. Future studies to further improve and validate the model’s performance in various forms of texts are warranted. We do not have evidence that users of the schizophrenia subreddit (r/schizophrenia) are clinically diagnosed. Similarly, users who participate in the schizophrenia subreddit are not necessarily representative of their population and are subject to selection bias [8]. Nevertheless, prior work has shown that individuals who participated in different mental health communities in Reddit are likely to be struggling from the type of their respective mental illnesses [16]. Social media data accompanied by formal clinical diagnoses are needed to provide a definitive identification of individuals with schizophrenia and their linguistic markers that will lead to early detection and diagnosis of schizophrenia [7]. We only evaluated commonly used machine learning classifiers while deep learning models have surpassed classical machine learning approaches in various text classification tasks such as semantic classification, sentiment analysis, question answering, and natural language inference [46]. Previous studies have proved that text classification methods based on deep learning algorithms, such as deep average networks (DAN) [47,48], long short-term memory (LSTM) [49,50], convolutional neural networks (CNN) [51,52], and graph neural networks (GNN) [53,54], outperform traditional machine learning methods when processing large-scale and complex datasets. Future work could assess advanced deep learning models, but at the cost of interpretability and model complexity. Finally, these findings may be limited to Reddit users, who may differ from individuals who use other social media platforms such as Twitter [6,17,19].

## 5. Conclusions

We collected a large corpus of schizophrenia posts from social media users and leveraged natural language processing and machine learning approaches to analyze and compare language from their posts and those of a control group comprising six non-mental health related subreddits. We demonstrated that our approach using machine learning methods and social media texts can be effectively used to detect signs of schizophrenia. Our findings indicate that distinctive linguistic patterns and content topics are present in individuals experiencing schizophrenia-related difficulties. In schizophrenia, we identified significant differences in linguistic features including increased use of third person plural pronouns and negative emotion words (e.g., anger, fear, sadness). Using unsupervised LDA clustering, we discovered the topics representing the major symptoms of schizophrenia including hallucinations, delusions, and negative symptoms. Importantly, we performed successful classification between the schizophrenia and the non-schizophrenia (control) groups with a highest accuracy of 96% on the basis of topic distributions and LIWC features. Our machine learning models proved effective even when trained with texts that did not contain the words “schizophrenia” or “schizo”. We further determined important features to identify language markers of schizophrenia in social media users. Finally, we established coherent semantic groups of words key to detecting schizophrenia, including symptom-related words (hallucinations, paranoia, delusions), negative sentiment words (fear, scare, afraid), and words related to mental health help (antipsychotic, research, profession). This study suggests that machine learning approaches combined with natural language processing could help understand the linguistic characteristics of schizophrenia and identity individuals with schizophrenia or otherwise at-risk individuals using social media texts.

## Figures and Tables

**Table 1:** Summary and description of the collected data from Reddit.

Group	Subreddit	Number of Posts	Description
Schizophrenia	Schizophrenia	13,156	Subreddit to discuss schizophrenia spectrum disorders and schizophrenia-related issues such as psychosis
Non-schizophrenia(Control)	Fitness	28,660	A place for the pursuit of physical fitness goals
	Jokes	83,456	The funniest subreddit (hundreds of jokes posted each day)
	Meditation	11,976	Community for sharing experiences, stories and instruction relating to the practice of meditation
	Parenting	23,489	A place to discuss the ins and out as well as ups and downs of child-rearing
	Relationships	97,038	Community built around helping people and the goal of providing a platform for interpersonal relationship advice between people
	Teaching	2950	A place to discuss news, recourses, and tips for teachers of all levels of education

**Table 2:** Pairwise t-test results of the linguistic features between the schizophrenia and the control groups.

LIWC Variable	t-Statistic	*p*-Value	LIWC Variable	t-Statistic	*p*-Value
**Linguistic processes**			**Psychological processes**		
Word count	−74.1	<0.001	Positive emotion	−4.7	<0.001
Words longer than six letters	53.7	<0.001	Negative emotion	30.0	<0.001
**Function words**			Anger	14.9	<0.001
Personal pronouns	−62.6	<0.001	Fear	47.6	<0.001
First person singular	0.02	0.981	Joy	−20.8	<0.001
First person plural	−76.5	<0.001	Disgust	8.8	<0.001
Second person	37.9	<0.001	Sadness	19.6	<0.001
Third person singular	−101.9	<0.001	Anticipation	−3.9	<0.001
Third person plural	24.3	<0.001	Trust	0.3	0.753
Impersonal pronouns	31.1	<0.001	Surprise	−0.5	0.616
**Time orientation**					
Past focus	−34.7	<0.001			
Present focus	24.4	<0.001			
Future focus	−0.04	0.965			

**Table 3:** Manually labeled topics and the top 10 words associated with each topic extracted from the schizophrenia and non-schizophrenia (control) subreddits.

Topics	Words
**Schizophrenia**	
Hallucinations	voice, hear, like, think, hallucinate, schizophrenia, thing, people, know, head
Delusions	feel, like, think, thing, know, time, delusion, experience, start, people
Mental health help	think, friend, know, help, tell, want, people, talk, say, like
Life	feel, want, like, know, time, life, go, think, year, tell
Medicine/Medication	medic, take, med, effect, month, doctor, antipsychotic, work, ability, experience
Schizophrenia/Diagnosis	schizophrenia, mental, help, diagnosis, experience, discord, symptom, know, thank, people
Family	year, help, time, mother, live, go, sister, house, work, family
Negative symptoms	negative, symptom, negative symptom, like, lack, schizophrenia, good, thing, work, motivation
Social interaction	people, like, know, want, go, thing, game, talk, feel, life
Thought disorder/Episode	go, think, tell, say, start, episode, week, walk, house, like
**Non-schizophrenia (Control)**	
Friend	friend, like, know, talk, tell, want, say, girl, think, feel
Feel/Relationship	feel, relationship, want, like, time, love, year, know, thing, think
Social/Life	tell, say, like, know, want, thing, feel, talk, go, time
Friend/Home/School	friend, go, time, drink, home, come, school, week, want, night
Family	parent, family, year, want, live, sister, work, mother, kid, husband
Joke	say, look, ask, walk, go, joke, like, come, tell, fuck
Meditation/Weight	meditate, weight, like, body, start, lose, eat, help, mind, calorie
Fitness/Exercise	workout, weight, week, work, exercise, lift, train, muscle, start, like
Time/Sleep	time, sleep, play, like, work, room, night, game, get, go
School/Teaching	school, teacher, teach, https, class, people, student, learn, read, book

Latent Dirichlet Allocation (LDA) reveals prominent topics in schizophrenia and non-schizophrenia (control) groups. The order of the topics indicates the average distribution of each topic, with Hallucinations being the highest and Thought disorder/Episode being the lowest for the schizophrenia group, as well as with Friend being the highest and School/Teaching the lowest for the control group. The order of the words in each topic indicates the order of word distribution within a topic.

**Table 4:** Classification performance of the machine learning classifiers.

Model	Recall	Precision	F1-Score	Accuracy	AUC
Random forest (RF)	0.94	0.98	0.96	0.96	0.97
Support vector machine (SVM)	0.91	0.90	0.91	0.91	0.91
Logistic regression (LR)	0.87	0.91	0.89	0.89	0.90
Naive Bayes (NB)	0.87	0.82	0.93	0.86	0.87

**Table 5:** Top 10 weighted features from the logistic regression classifier for the schizophrenia and control groups.

Feature	Weight
Symptom	1.12
Voice	1.00
Diagnose	0.88
Psychotic	0.72
Delusions	0.71
Hallucinations	0.68
Paranoia	0.67
Medication	0.65
Psychosis	0.61
Medicine	0.55

**Table 6:** Lists of the most predictive words for schizophrenia.

Cluster Color	Semantic Content	Words in Cluster
Orange	Symptoms/Hallucinations	Symptom, voice, diagnosis, hallucination, medic, hear
Yellow	Symptoms/Psychosis	psychotic, paranoia, med, psychosis, episode, psychiatrist, paranoid, discord, diagnosis, heard, hospital, brain, reality, drug
Red	Delusions/Suffering	delusion, suffer, hi, hurt, didn’t
Blue	Fear	fear, people, scare, thought, remember, afraid, believe, actual, understand, life, run, mind, worry, kill, certain, head, sense, watch, action, think, beauty, twice, connect, sudden, manipulate, happen, go
Pink	Prognosis	tomorrow, insane, attempt, safe, fake, free, disable, isolate, patient, stuck, odd
Purple	Support	abilify, antipsychotic, research, delusion, condition, medicine, psych, profession, god, strange, trigger, attack, sub, music, anybody, form
Black	Present	shit, wish, took, today, alone, state, hell, anyway
Green	Mental health help	mental, anyone, get, ill, ani, read, else, help, thank, everyone, wonder, experience

The words are organized in the clusters labeled by their color in Figure 6, and by the general semantic content of the words in schizophrenia. The order of the clusters indicates the average predictiveness of the cluster of schizophrenia, with Symptoms/Hallucinations being the most predictive, and the Mental health help cluster being the least predictive. The order of the words in each cluster indicates the order of predictiveness within a cluster.

## Data Availability

The datasets generated during the current study are available from the corresponding author on reasonable request. The code supporting this paper is available at https://github.com/khu-aims/schizophrenia-detection (accessed on 29 August 2021).

## Supplementary Materials

The following are available online at https://www.mdpi.com/article/10.3390/s21175924/s1, Figure S1: histogram of post lengths as measured by the length of tokens for each subreddit, Figure S2: algorithm performance based on the input features entered in the model, Figure S3: text plot of words that distinguish the language of the schizophrenia group from the non-schizophrenia (control) group, Figure S4: text plot of words that distinguish the language of the schizophrenia group from the non-schizophrenia (control) group using the first half of the posts, Figure S5: text plot of words that distinguish the language of the schizophrenia group from the non-schizophrenia (control) group using the second half of the posts, Table S1: summary of the collected data from Reddit, Table S2: classification performance of the machine learning classifiers, Table S3: ranked list of the features with the mean absolute Shapley value (descending order), Table S4: lists of the most predictive words for schizophrenia, Table S5: lists of the most predictive words for schizophrenia using the first half of the posts, Table S6: lists of the most predictive words for schizophrenia using the second half of the posts.
