# Supplementary material for: Schizophrenia Detection Using Machine Learning Approach from Social Media Content
Source: Sensors (Basel). 2021 Sep 3;21(17):5924. doi: 10.3390/s21175924 (PMC8434514; doi:10.3390/s21175924)
Supplement: Supplementary file 1 [file sensors-21-05924-s001.zip › sensors-1340970_supplementary_proofread_figures.pdf]

Supplementary Materials for Schizophrenia detection using machine learning approach from social media content

1. Dataset

Supplementary Table 1 summarizes the information of the collected data from Reddit.

Table S1. Summary of the collected data from Reddit.

Group	Subreddit	Number of posts	Number of tokens	Mean (SD) post length	Mean (SD) token length
Schizophrenia	Schizophrenia	13,156	776,204	326.07 (360.56)	5.59 (1.64)
Non-schizophrenia (Control)	Fitness	28,660	1,340,959	252.62 (275.97)	5.39 (1.61)
	Jokes	83,456	1,229,411	79.12 (187.35)	5.37 (1.66)
	Meditation	11,976	633,322	286.21 (401.98)	5.41 (1.56)
	Parenting	23,489	1791,109	403.56 (372.02)	5.29 (1.53)
	Relationships	97,038	15,217,239	828.91 (631.21)	5.28 (1.63)
	Teaching	2950	173,895	329.66 (371.66)	5.59 (1.64)

SD = standard deviation

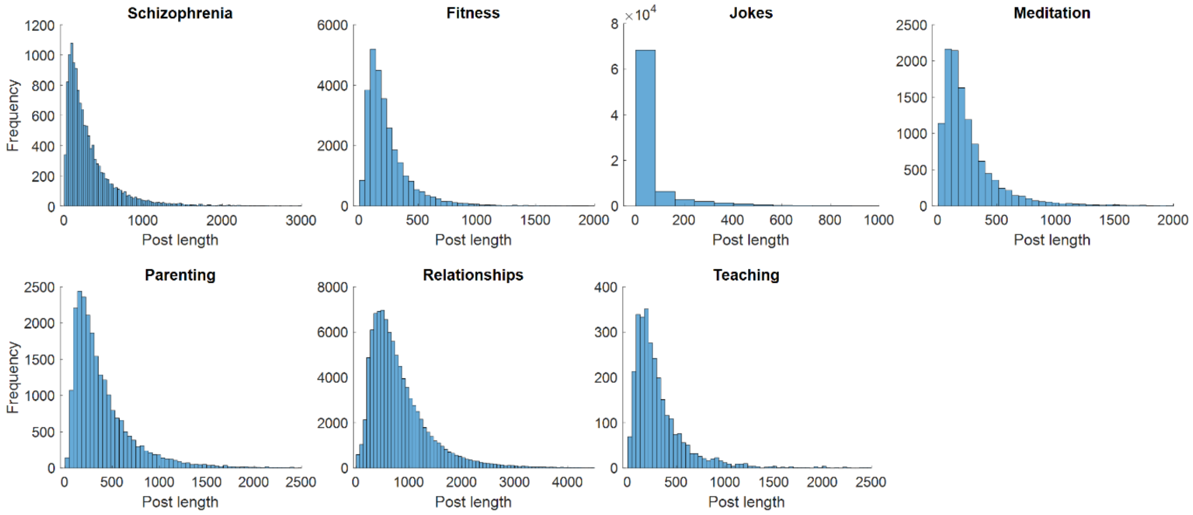

Figure S1. Histogram of post lengths as measured by the length of tokens for each subreddit.

2. Comparison of Algorithm Performance Based on the Input Features

All models performed better when all features (LIWC and topic distributions) were included in the model; therefore, all subsequent analyses were based only on these models with all combined features. The random forest algorithm consistently outperformed all the other algorithms regardless of the type of input data (Supplementary Table S2; Supplementary Figure S2).

Table S2. Classification performance of the machine learning classifiers.

Feature set	Model	Recall	Precision	F1-score	Accuracy	AUC
LIWC	Random forest (RF)	0.92	0.98	0.95	0.95	0.95
	Support vector machine (SVM)	0.88	0.88	0.88	0.88	0.88
	Logistic regression (LR)	0.85	0.85	0.85	0.85	0.85
	Naive Bayes (NB)	0.80	0.81	0.80	0.80	0.81
Topic	Random forest (RF)	0.92	0.97	0.94	0.95	0.95
	Support vector machine (SVM)	0.93	0.94	0.93	0.90	0.94
	Logistic regression (LR)	0.82	0.88	0.85	0.85	0.86
	Naive Bayes (NB)	0.80	0.81	0.80	0.79	0.84
Joint	Random forest (RF)	0.94	0.98	0.96	0.96	0.97
	Support vector machine (SVM)	0.91	0.90	0.91	0.91	0.91
	Logistic regression (LR)	0.87	0.91	0.89	0.89	0.90
	Naive Bayes (NB)	0.87	0.82	0.93	0.86	0.87

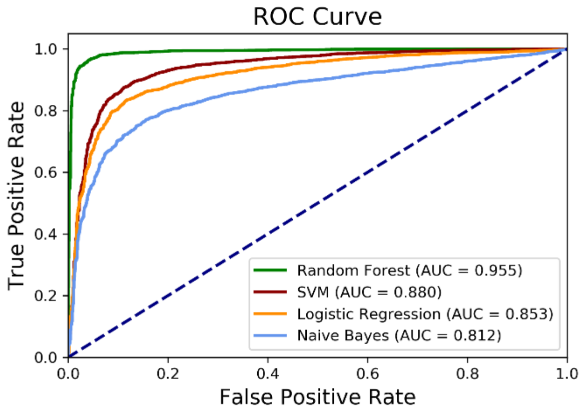

(a)

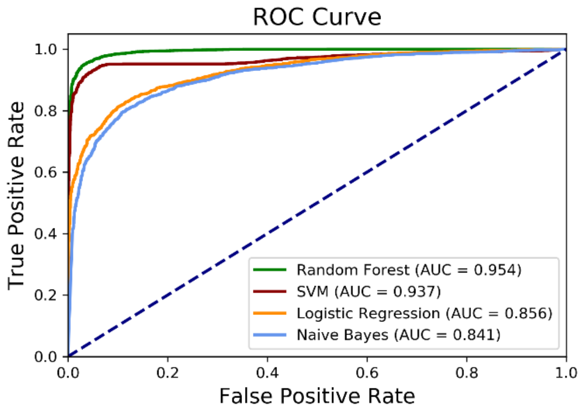

(b)

Figure S2. Algorithm performance based on the input features entered in the model. Shown are the receiver operating characteristic (ROC) curves for the classification based on (a) LIWC features alone and (b) topic features alone. Additional details are provided in Supplementary Table S2.

3. Feature Importance

By applying the SHAP approach to the random forest model, we estimated the feature importance to determine features key to distinguishing schizophrenia posts from

control posts. The SHAP feature importance quantified as the mean absolute Shapley value for all features is listed in Supplementary Table S3.

Table S3. Ranked list of the features with the mean absolute Shapley value (descending order).

Ranking	Feature	Mean absolute Shapley value	Ranking	Feature	Mean absolute Shapley value
1	Third person singular	0.106037	22	Fitness/Exercise (Non-SCZ topic)	0.006893
2	Third person plural	0.077902	23	Feel/Relationship (Non-SCZ topic)	0.006732
3	Meditation/Weight (non-SCZ topic)	0.053068	24	Third person plural	0.006570
4	Hallucinations (SCZ topic)	0.045677	25	Social interaction (SCZ topic)	0.005944
5	Friend (non-SCZ topic)	0.045103	26	Joke (non-SCZ topic)	0.005077
6	Mental health help (SCZ topic)	0.044755	27	Friend/Home/School (non-SCZ topic)	0.004546
7	School/Teaching (non-SCZ topic)	0.031163	28	Impersonal pronouns	0.004194
8	Life (SCZ topic)	0.030233	29	Second person	0.003761
9	Word count	0.028808	30	Thought disorder/Episode (SCZ topic)	0.003356
10	Fear	0.026922	31	Present focus	0.003088
11	Social/Life (non-SCZ topic)	0.021443	32	Negative emotion	0.002936
12	Time/Sleep (non-SCZ topic)	0.015646	33	Anticipation	0.002657
13	Schizophrenia/Diagnosis (SCZ topic)	0.014710	34	Positive emotion	0.002326
14	Words longer than six letters	0.013840	35	Trust	0.001914
15	First person singular	0.013036	36	Disgust	0.001722
16	Personal pronouns	0.010836	37	Negative symptoms (SCZ topic)	0.001681
17	Family (SCZ topic)	0.010804	38	Surprise	0.001636
18	Medicine/Medication (SCZ topic)	0.009328	39	Sadness	0.001586
19	Joy	0.008279	40	Past focus	0.001384
20	Family (non-SCZ topic)	0.007345	41	Anger	0.001291
21	Delusion (SCZ topic)	0.006944	42	Future focus	0.001224

3. Reliability Analyses

3.1. Reliability of Unsupervised Clustering Results Using TF-IDF Features with Bigrams

In the main manuscript, we showed unsupervised clustering results using TF-IDF features with unigrams (i.e., a sequence of one word in a sentence). To test the reliability of the unsupervised clustering results, we repeated the analyses using TF-IDF features with bigrams (i.e., a sequence of two words in a sentence). The results remained consistent when an alternative bigram vectorization technique along with TF-IDF features was used to identify language markers of schizophrenia (Supplementary Figure S3 and Table S4).

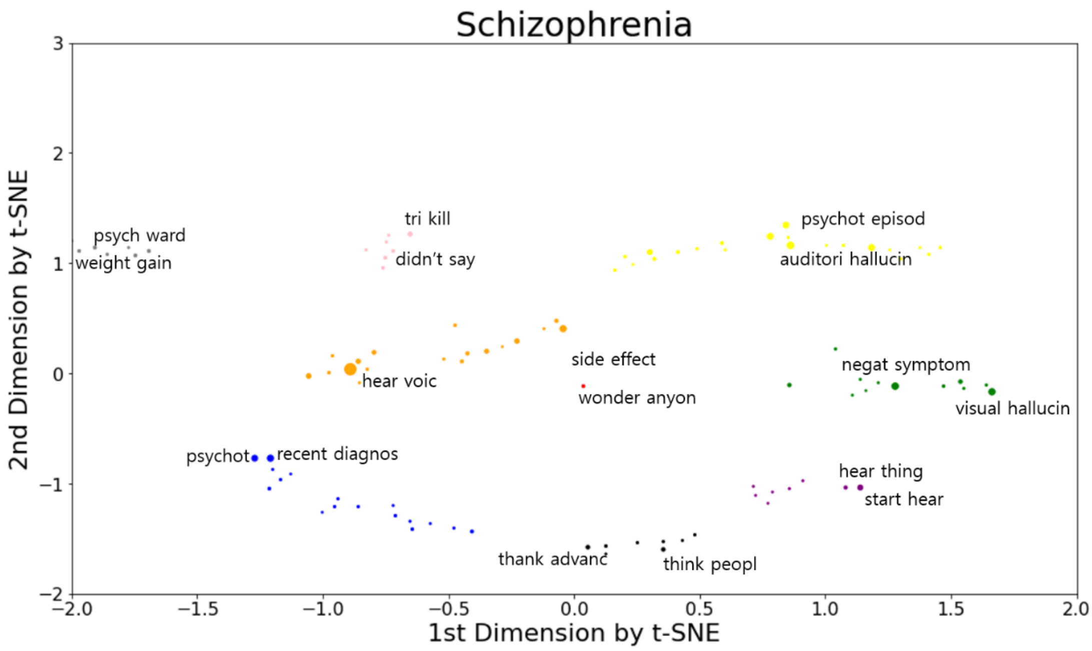

Figure S3. Text plot of words that distinguish the language of the schizophrenia group from the non-schizophrenia (control) group. The 100 most predictive words for schizophrenia derived using TF-IDF features with bigrams. Words are projected in a 2D space based on their document vectors, after dimensionality reduction with t-SNE. Colors indicate clusters assigned by DBSCAN. In each cluster, the top two most predictive words are labeled.

Table S4. Lists of the most predictive words for schizophrenia.

Cluster color	Semantic content	Words in cluster
Orange	Symptoms/Hallucinations	hear voic, side effect, anyon els, get help, mental ill, get wors, mental health, see thing, im scare, make sens, doe anyon, see peopl, sound, go back, even know, thing like, would say, im afraid
Yellow	Symptoms/Psychosis	psychot episod, auditori hallucin, take medic, voic head, take med, smoke weed, think might, sometim feel, thing happen, go away, start think, im sorr, becaus thought, need get, panic attack, would go, onli thing, tri help, thank read, dure time, like feel
Red	Help	wonder anyon, time befor
Green	Negative symptoms	negat symptom, visual hallucin, stop take, peopl talk, talk peopl, anyon know, peopl know, sinc ive, fall asleep, start say, wish could, im onli
Purple	Symptoms/Hearing	start hear, hear thing, live life, could tell, four year, first start, ago start, make ani
Pink	Life problems	tri kill, didnt say, hi everyon, feel free, anyon ever, becaus hes, intrus thought
Black	Support/Advice	thank advanc, think peopl, love one, see doctor, veri long, would great, great appreci, see therapist
Gray	Health	weight gain, psych ward, like everyon, doesnt make, person disord, like even, know anyth

The words are organized in the clusters labeled by their color in Supplementary Figure S3, and by the general semantic content of the words in schizophrenia. The order of the clusters indicates the average predictiveness of the cluster of schizophrenia, with Symptoms/Hallucinations being the most predictive, and the Health cluster being the least predictive. The order of the words in each cluster indicates the order predictiveness within a cluster.

3.2. Reliability of Unsupervised Clustering Results Using Split-Half Datasets

To test the reliability of the unsupervised clustering results, we created split-half datasets by randomly resampling half of the posts and then performed the classification tasks on the split-half datasets 100 times. Using a number of split-half post datasets, the logistic regression classifiers achieved predictive accuracies (96–98%) for classification of schizophrenia posts similar to those with the original full datasets used in the main manuscript. We then repeated the same unsupervised clustering analyses on the regression weights estimated from half of the sample. The results remained consistent when analyses were restricted only to half of the posts. We provide representative clustering results from the first half of the posts (Supplementary Figure S4 and Table S5) and the second half of the posts (Supplementary Figure S5 and Table S6).

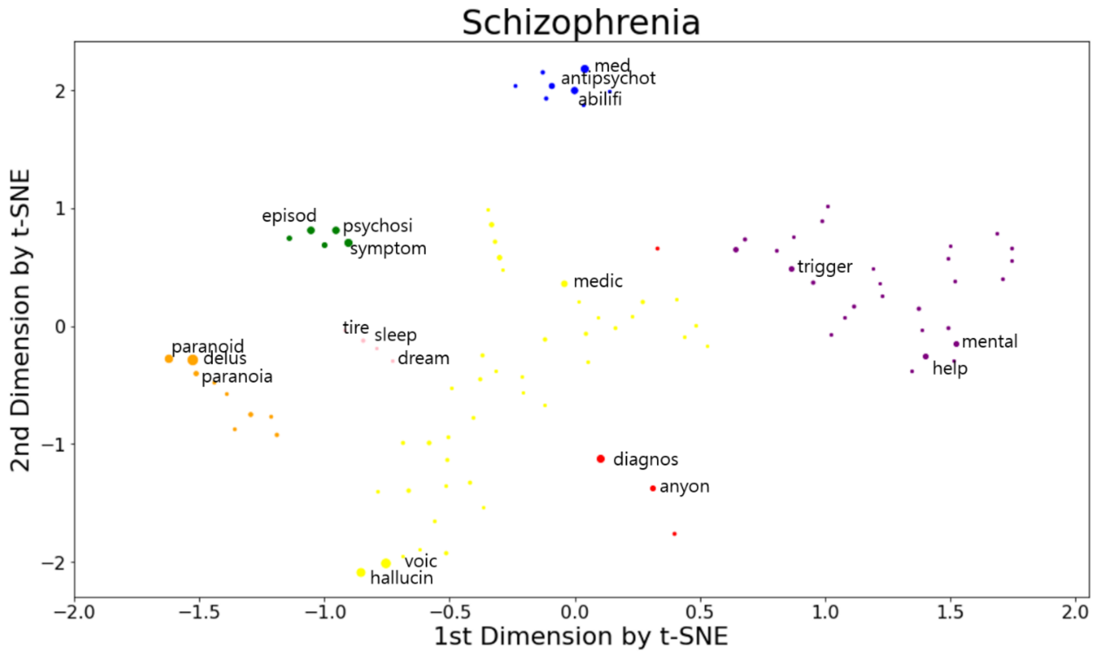

Figure S4. Text plot of words that distinguish the language of the schizophrenia group from the non-schizophrenia (control) group using the first half of the posts. The 100 most predictive words for schizophrenia using the first half of the posts. Words are projected in a 2D space based on their document vectors, after dimensionality reduction with t-SNE. Colors indicate clusters assigned by DBSCAN. In each cluster, the top three most predictive words are labeled.

Table S5. Lists of the most predictive words for schizophrenia using the first half of the posts.

Cluster color	Semantic content	Words in cluster
Purple	Mental health help	help, mental, trigger, experi, insight, understand, wonder, hello, ill, live, hope, suffer, curious, post, support, job, pleas, univers, inform, spoke, ani, veri, communiti, char-act, member, public, found, comment
Orange	Delusions	delus, paranoid, paranoia, realiti, real, mind, delusion, peopl, harm
Yellow	Symptoms/Hallucinations	voic, hallucin, medic, hospit, psychiatrist, scare, feel, any-bodi, psych, ago, think, shower, demon, talk, im, develop, stare, havent, normal, shadow, treatment, weird, someth, degre, vent, go, brain, appoint, bare, everyon, god, mes-sag, function, exact, slowli, sometim, wish, random, face, medicin, head
Green	Psychosis/Disorder	symptom, episod, psychosi, psychot, disord
Blue	Medicine/Medication	med, abilifi, antipsychot, dose, effect, prescrib, gain, pill
Red	Diagnose/Diagnosis	diagnos, diagnosi, anyon, anymor, els
Pink	Sleep issues	sleep, dream, tire, idk

The words are organized in the clusters labeled by their color in Supplementary Figure S4, and by the general semantic content of the words in schizophrenia. The order of the clusters indicates the average predictiveness of the cluster of schizophrenia, with Mental health help being the most predictive, and the Sleep issues cluster being the least predictive. The order of the words in each cluster indicates the order predictiveness within a cluster.

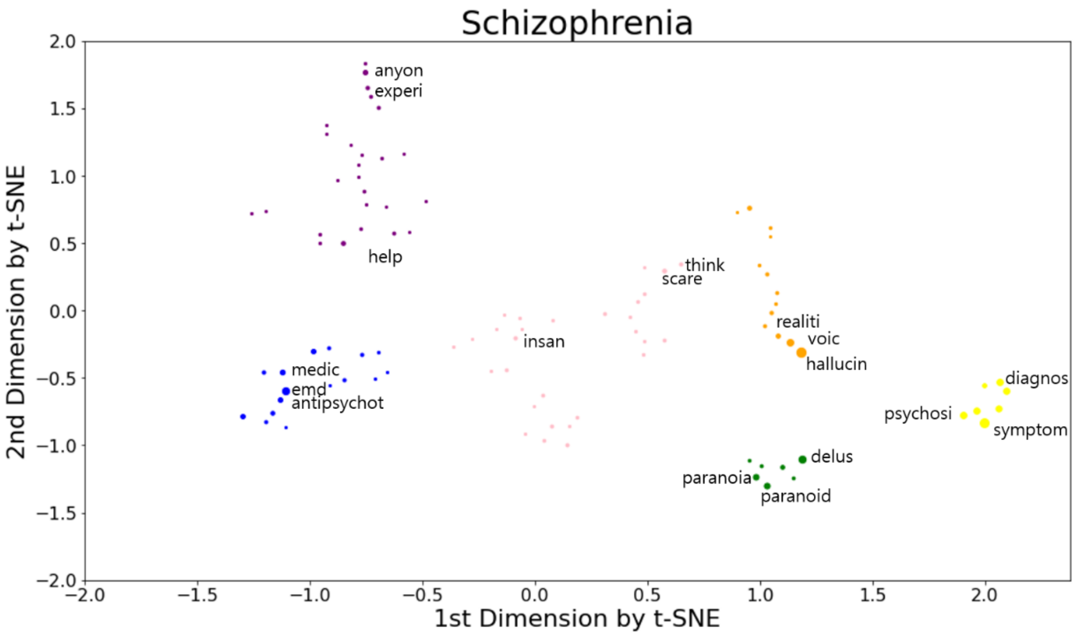

Figure S5. Text plot of words that distinguish the language of the schizophrenia group from the non-schizophrenia (control) group using the second half of the posts. The 100 most predictive words for schizophrenia using the second half of the posts. Words are projected in a 2D space based on their document vectors, after dimensionality reduction with t-SNE. Colors indicate clusters assigned by DBSCAN. In each cluster, the top three most predictive words are labeled.

Table S6. Lists of the most predictive words for schizophrenia using the second half of the posts.

Cluster color	Semantic content	Words in cluster
Yellow	Symptom/Psychosis	symptom, psychosi, diagnos, episod, diagnosi, psychot, disord
Green	Delusions	delus, paranoid, paranoia, delusion, ocd, believ, sign
Red	Mental illness	ill, mental
Blue	Medicine/Medication	med, medic, antipsychot, hospit, abilifi, prescrib, psychiatrist, psych, medicin, effect, treatment, function, pill, caus, appoint, gain
Orange	Symptoms/Hallucinations	hallucin, voic, realiti, im, real, sometim, sens, head, feel, someth, thought, like, dont
Purple	Mental health help	anyon, help, experi, relat, job, wonder, stori, support, famili, subreddit, communiti, els, pleas, hello, inform, ani, danger, condit, disabl, messag, affect, wish, hi, suffer, brother, today
Pink	Life problems	scare, insan, think, demon, shadow, anybodi, someon, talk, brain, stare, anymor, ago, trigger, peop', understand, outsid, mayb, life, everyon, sleep, memori, god, random, idk, sort, odd, dream, shower, normal

The words are organized in the clusters labeled by their color in Supplementary Figure S5, and by the general semantic content of the words in schizophrenia. The order of the clusters indicates the average predictiveness of the cluster of schizophrenia, with Symptoms/Psychosis being the most predictive, and the Life problems cluster being the least predictive. The order of the words in each cluster indicates the order predictiveness within a cluster.
